# The membrane mucin Msb2 regulates aflatoxin biosynthesis and pathogenicity in fungus *Aspergillus flavus*

**DOI:** 10.1111/1751-7915.13701

**Published:** 2020-11-07

**Authors:** Ling Qin, Ding Li, Jiaru Zhao, Guang Yang, Yinchun Wang, Kunlong Yang, Elisabeth Tumukunde, Shihua Wang, Jun Yuan

**Affiliations:** ^1^ Key Laboratory of Pathogenic Fungi and Mycotoxins of Fujian Province Key Laboratory of Biopesticide and Chemical Biology of Education Ministry School of Life Sciences Fujian Agriculture and Forestry University Fuzhou 350002 China

## Abstract

As a pathogenic fungus, *Aspergillus flavus* can produce carcinogenic aflatoxins (AFs), which poses a great threat to crops and animals. Msb2, the signalling mucin protein, is a part of mitogen‐activated protein kinase (MAPK) pathway which contributes to a range of physiological processes. In this study, the roles of membrane mucin Msb2 were explored in *A. flavus* by the application of gene disruption. The deletion of *msb2* gene (Δ*msb2*) caused defects in vegetative growth, sporulation and sclerotia formation when compared to WT and complement strain (Δ*msb2^C^*) in *A. flavus*. Using thin‐layer chromatography (TLC) and high‐performance liquid chromatography (HPLC) analysis, it was found that deletion of *msb2* down‐regulated aflatoxin B_1_ (AFB_1_) synthesis and decreased the infection capacity of *A. flavus*. Consistently, Msb2 responds to cell wall stress and osmotic stress by positively regulating the phosphorylation of MAP kinase. Notably, Δ*msb2* mutant exhibited cell wall defect, and it was more sensitive to inhibitor caspofungin when compared to WT and Δ*msb2^C^*. Taking together, these results revealed that Msb2 plays key roles in morphological development process, stresses adaptation, secondary metabolism and pathogenicity in fungus *A. flavus*.

## Graphical abstract

Msb2 is important for growth, conidia and sclerotia formation in *Aspergillus flavus*. Msb2 play negative roles in regulation of aflatoxins biosynthesis.

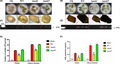

## Introduction

*Aspergillus flavus* is a famous plant pathogenic fungus, which is notorious as the main producer of aflatoxins (AFs) (Amaike and Keller, 2011). *A. flavus* can contaminate many agricultural crops (such as maize, peanut, cotton and so on) causing huge economic losses (Wu *et al*., 2014). Studies have shown that food contaminated by low concentration of AFs may lead to hepatocellular carcinoma, while high concentration of AFs can be toxic, and in some cases could be fatal (Amare and Keller, 2014). A 70 kb gene cluster was identified for AFs biosynthesis which encodes about 25 enzymes and other key regulatory proteins in *A. flavus* (Bhatnagar *et al*., 2003). Previous studies have demonstrated that internal and external factors controlled the AF biosynthesis, such as developmental stage and hyperosmolarity (Tsitsigiannis and Keller, 2006; Zhang *et al*., 2018a, 2018b,2018a, 2018b). These outcomes have indicated that an appropriate respond to environmental changes is essential for AF biosynthesis in *A. flavus*.

Environmental stimuli beyond the physiological range can be a matter of life or death for all cells (Aguilera, 2002). Mitogen‐activated protein kinases (MAPKs) cascades are evolutionarily conserved signalling units that are utilized for signal transduction in diverse eukaryotic organisms (Chen *et al*., 2001). Five main MAPK signalling pathways have been identified and characterized in *Saccharomyces cerevisiae*, including the high osmolarity glycerol (HOG) pathway, the mating response pathway, filamentous and invasive growth (FIG) pathway, cell wall integrity (CWI) pathway and pheromone pathway (Saito and Posas, 2012). But in *A. flavus*, only three central MAP kinases orthologous to yeast, including SakA (Hog1), Slt2 (MPKA) and Fus3 (MPKB), have been described (Tumukunde *et al*., 2019; Zhang *et al*., 2020; Frawley *et al*., 2020). These MAP kinases have distinct functions in asexual sporulation, sclerotia formation and aflatoxin production. However, each MAPK module is activated by specific types of stimuli and received signalling by specific sensors (Free, 2013; Tanaka *et al*., 2014; Chow *et al*., 2019).

Signalling mucin protein Msb2, as one of the putative osmosensor in *S*. *cerevisiae*, initiates signalling response in the HOG pathway (Tatebayashi *et al*., 2007). Interestingly, the Msb2 homologue protein MsbA in *Aspergillus fumigatus* exhibits different functions, which are essential for stresses adaptation, and in resistance to antifungal drugs through modulating the gene expression of the CWI pathway (Gurgel *et al*., 2019). Also, the signalling mucin Msb2 protein activates the CEK1‐MAPK pathway in human fungal pathogen *Candida albicans* (Puri *et al*., 2012), Pmk1‐MAPK (homologous to Fus3 in *S. cerevisiae*) pathway in the rice blast *Magnaporthe oryzae* (Wang *et al*., 2015), and the CWI pathway in *Aspergillus nidulans* (Brow *et al*., 2014). Moreover, Msb2 protein has been extensively characterized as an external sensor activating Fmk1, a MAP kinase in FIG pathway, for cell growth in *Fusarium oxysporum* (Perez‐Nadales and Di Pietro, 2015).

Although the roles of Msb2 have been addressed in multiple filamentous fungi, such as involving in plant infection in *F. oxysporum* and *Ustilago maydis*, appressorium formation in *M. oryzae*, and hyphal growth in *Histoplasma capsulatum* (Pérez‐Nadales and Di Pietro, 2011; Wang *et al*., 2015; Rodriguez *et al*., 2019), the function of this protein in *A. flavus* was poorly understood. In this study, *Aflmsb2* deletion and complement mutants were generated, and the results demonstrated that AflMsb2 functioned in fungal growth, asexual development and sclerotia formation in *A. flavus*. The results also showed that Aflmsb2 was crucial for osmotic adaptation and cell wall integrity. Notably, AflMsb2 was involved in keeping chitinase activity of *A. flavus* and contributed to the caspofungin resistance. Furthermore, this study also provided a first time report of the relationship between mucin protein AflMsb2 and aflatoxin biosynthesis in *A. flavus*.

## Results

### Identification and analysis of Msb2 in *A. flavus*

**Fig. 1:**
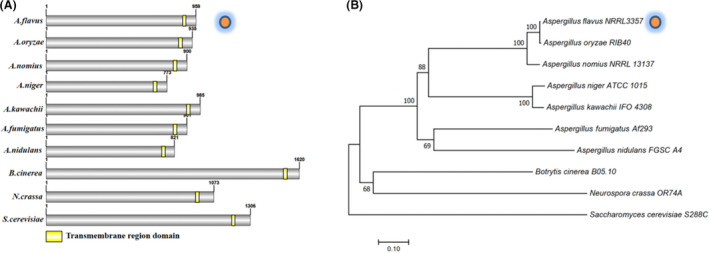
Bioinformatics analysis of the membrane mucin Msb2 proteins from 10 different fungi: *A. flavus* (XP_002385498.1), *A. oryzae* (OOO03851.1), *A. nomious* (XP_015401032.1), *A. niger* (XP_001400709.1), *A. kawachii* (GAA_87196.1), *A. fumigatus* (XP_746593.1), *A. nidulans* (EAA61687.1), *B. cinerea* (B0510_3063T1.1), *N. crassa* (XM_951053.3) and *S. cerevisiae* (NP_011528.3). A. Domains from Msb2 proteins were identified using online tools SMART and visualized by dog2.0 software. B. Phylogenetic relationship of Msb2 in the selected fungi was analysed and visualized by mega7.0.

To identify orthologs of the *S. cerevisiae* Msb2 in *A. flavus*, the protein sequence of *S. cerevisiae* (NP_011528.3) was used with a basic local alignment search tool algorithm (BLAST), and an orthologs gene was identified in *A. flavus* named *Aflmsb2* (AFLA_114450). *Aflmsb2* was predicted to encode 959 amino acids that contains a conserved transmembrane region domain at the C‐terminal (Fig. 1A). The phylogenetic analysis of signalling mucin Aflmsb2 showed a high conservation to Msb2 orthologue proteins from different fungi (Fig. 1B). Among them, Msb2 protein sequence of *A. flavus* exhibited the strongest similarity to that of *A. oryzae* which has been identified as a transmembrane mucin.

### Msb2 is involved in vegetative growth

**Fig. 2:**
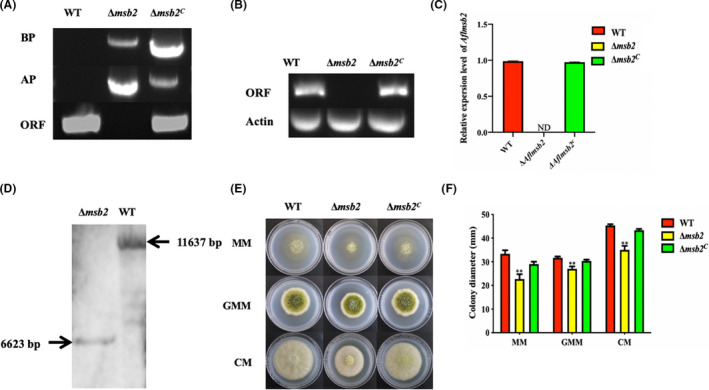
Construction of the deletion (Δ*msb2*) and complement (Δ*msb2^C^*) mutant strains. A. Verification of Δ*msb2* and Δ*msb2^C^* mutant strains by PCR, using gDNA as template. B. Confirmation of Δ*msb2* and Δ*msb2^C^* mutant strains by RT‐PCR, using cDNA as template. C. Expression levels of *msb2* in WT, Δ*msb2* and Δ*msb2^C^* mutant strains detected by qRT‐PCR. D. Confirmation of the deletion mutant by Southern blot. E. WT, Δ*msb2* and Δ*msb2^C^* strains were grown on MM, GMM and CM plates, respectively, for 4 days. F. Colony diameter of all the strains as in E was assayed. ND = Not detectable. **, denotes *P* < 0.01.

To evaluate the function of *Aflmsb2* in *A. flavus*, *Aflmsb2* deletion (Δ*msb2*) and complement (Δ*msb2^C^*) mutants were constructed by homologous recombination. The transformants were confirmed by PCR analysis with three pairs of primers, and the results showed that the AP and BP fragments were contained in both Δ*msb2* and Δ*msb2^C^* mutant strains, but absent in wild type (WT), indicating that the *A. fumigatus pyrG* gene had completely replaced the *msb2* gene in *A. flavus* (Fig. 2A). Expression levels of *msb2* in the WT, Δ*msb2* and Δ*msb2^C^* were confirmed by RT‐PCR and qRT‐PCR respectively, and the results shown in Figure 2B and C proved that *Aflmsb2* gene was existed in the WT and Δ*msb2^C^*, but was missing in Δ*msb2*. Ultimately, the deletion strain was further verified by Southern blot, which indicated that *Aflmsb2* was successfully disrupted in the mutants (Fig. 2D). Colony morphology was observed after incubation at 37°C in dark for 4 days, and the result indicated a reduction in the growth rate of the Δ*msb2* strains as compared to the WT and Δ*msb2^C^* strains in minimal medium (MM), complete medium (CM) and glucose minimal medium (GMM) (Fig. 2E and F), suggesting a defect in vegetative growth.

### Msb2 is involved in conidiation

**Fig. 3:**
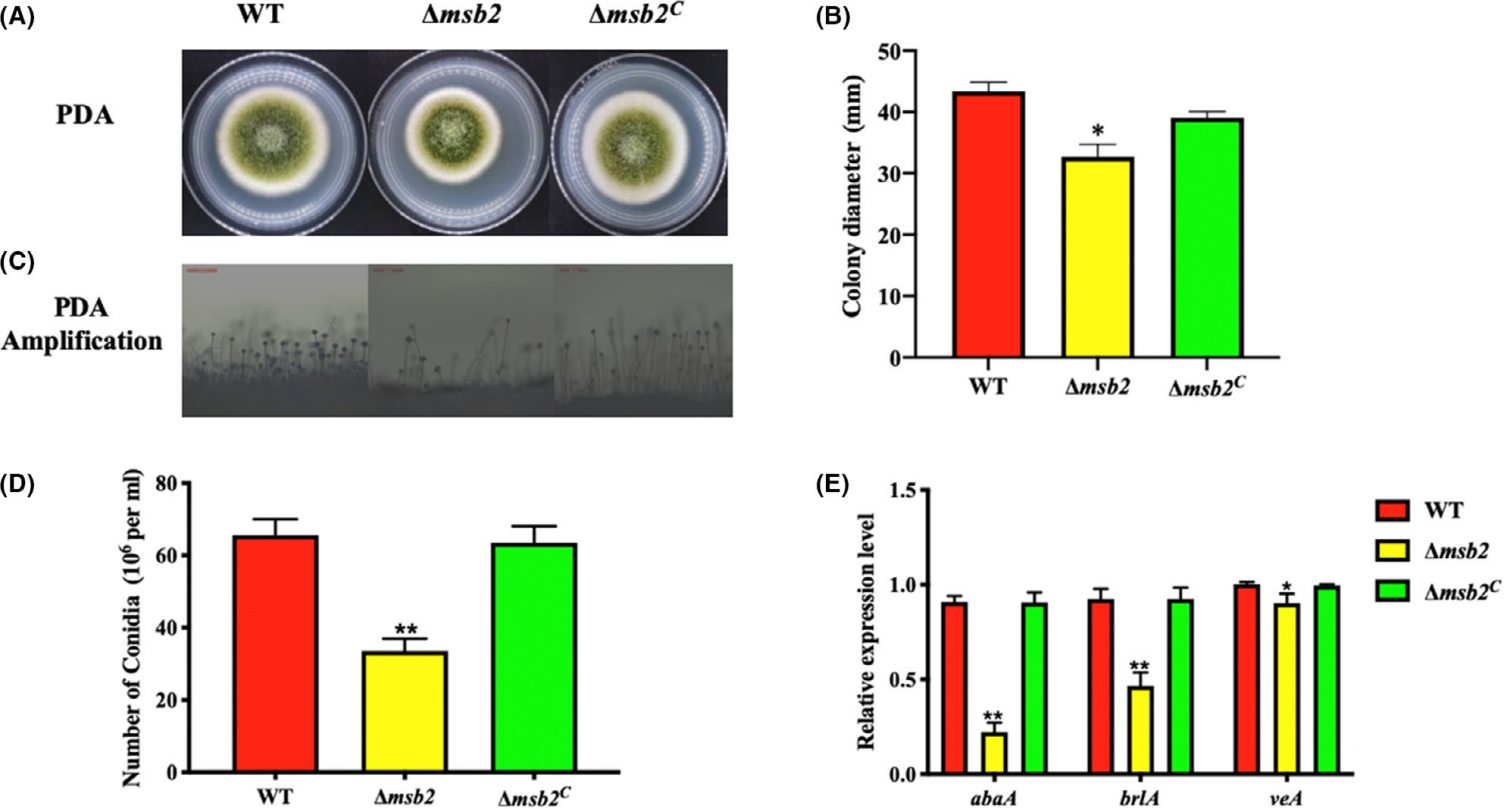
Phenotype and conidia production of strains. A. WT, Δ*msb2* and Δ*msb2^C^* strains of *A. flavus* were grown on PDA plates for 4 days. B. Colony diameter in PDA medium was assayed. C. Microscopic observation of conidiophores after 12 h incubation on PDA medium. D. Conidia production of WT, Δ*msb2* and Δ*msb2^C^* strains. E. qRT‐PCR analysis of the expression levels of genes *abaA*, *brlA* and *veA* among WT, Δ*msb2* and Δ*msb2^C^* strains. Bars = 200 μm. * and ** denotes *P* < 0.05 and *P* < 0.01, respectively.

Conidia is produced at the hypha tip, which is one of the most important asexual reproduction in *A. flavus* (Amaike and Keller, 2011). For analysis of the function of Msb2 in conidiation, the WT and mutant strains were grown on PDA medium for 4 days. Following microscopic observations, Δ*msb2* strain exhibited a growth reduction on PDA medium plates (Fig. 3A and B) and produced less and shorter conidiophores in comparison to the WT and Δ*msb2^C^* mutant strains (Fig. 3C). With respect to defect in conidiophores, we also found that the number of conidia was significantly decreased in Δ*msb2* mutant, as compared to the WT and Δ*msb2^C^* strains (Fig. 3D). We further studied the expression levels of regulatory genes for conidial formation (*brlA* and *abaA*) and global regulator gene (*veA*), and the result showed that the expression levels of *brlA*, *abaA* and *veA* genes were all down‐regulated in the Δ*msb2* strain in comparison to Δ*msb2^C^* and WT strains (Fig. 3E). All above results demonstrated that AflMsb2 is involved in conidial formation and vegetative growth in *A. flavus*.

### Msb2 positively regulates sclerotia formation in *A. flavus*

**Fig. 4:**
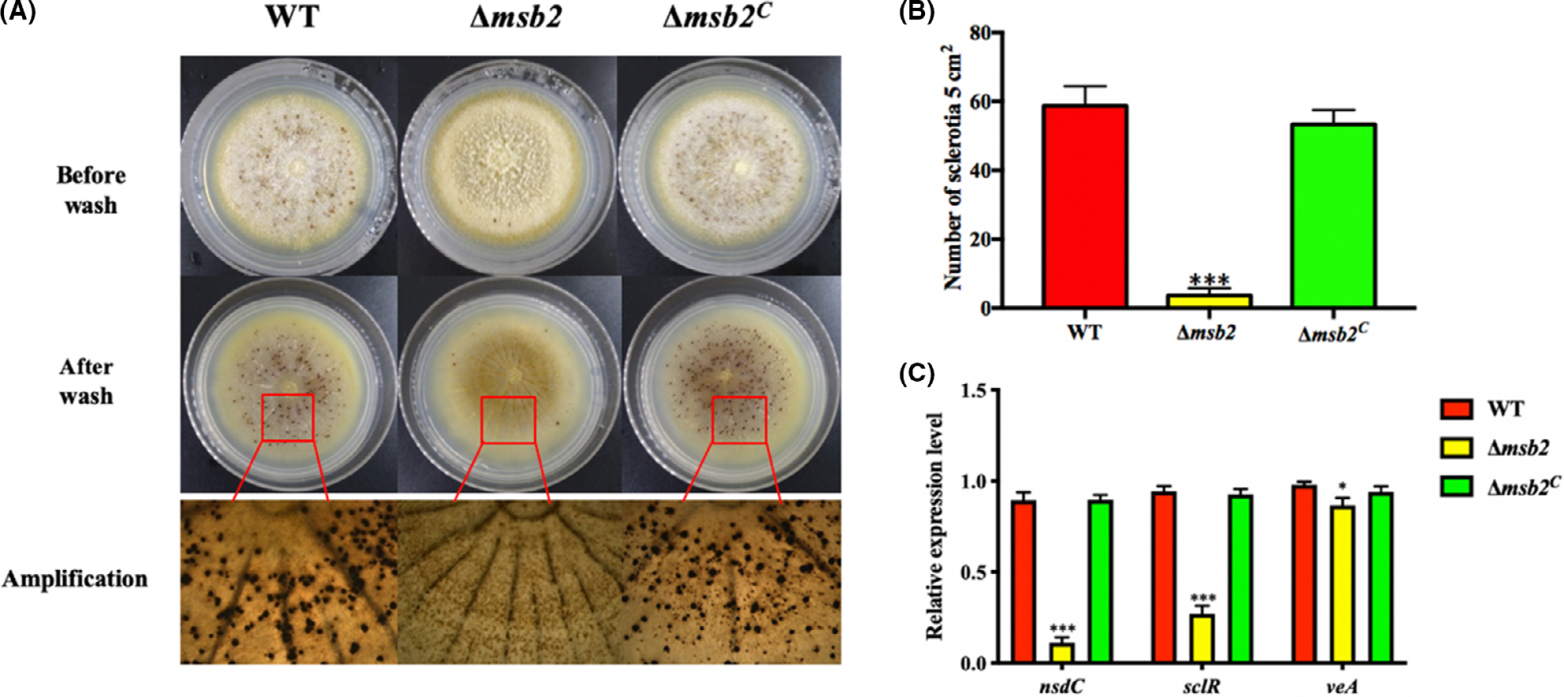
Phenotype analysis of *msb2* on sclerotia formation. A. Phenotypes of WT, Δ*msb2* and Δ*msb2^C^* grown on WKM plates in dark condition for 7 days. B. The number of sclerotia production in WT, Δ*msb2* and Δ*msb2^C^* strains respectively. C. The expression levels of sclerotia production related genes (*sclR* and *nsdC*) and global regulator gene (*veA*) among WT, Δ*msb2* and Δ*msb2^C^* strains, respectively. * and *** denotes *P* < 0.05 and *P* < 0.001, respectively.

Different to conidia, sclerotium is one of the alternative reproduction to survive in the adverse environments in *A. flavus* (Dyer and O'Gorman, 2012). To validate the function of Msb2 in formation of sclerotium, WT, Δ*msb2* and Δ*msb2^C^* strains were cultured on sclerotia‐inducing WKM solid medium, at 37℃ under dark condition for 7 days. As shown in Figure 4A and B, Δ*msb2* mutant barely able to generate sclerotium, whereas WT and Δ*msb2^C^* could produce similar amounts of sclerotia. In addition, we had detected the expression levels of *nsdC* and *sclR* genes, which are indispensable for sclerotia generation (Cary *et al*., 2012). As expected, the transcript levels of *sclR* and *nsdC* were both declined sharply in the Δ*msb2* strain when compared to Δ*msb2^C^* and WT strains, and the expression level of global regulator *veA* gene had also decreased (Fig. 4C). All these observed results strongly indicated that Msb2 plays a key role in sclerotia production of *A. flavus*.

### Effect of *msb2* on aflatoxin biosynthesis

**Fig. 5:**
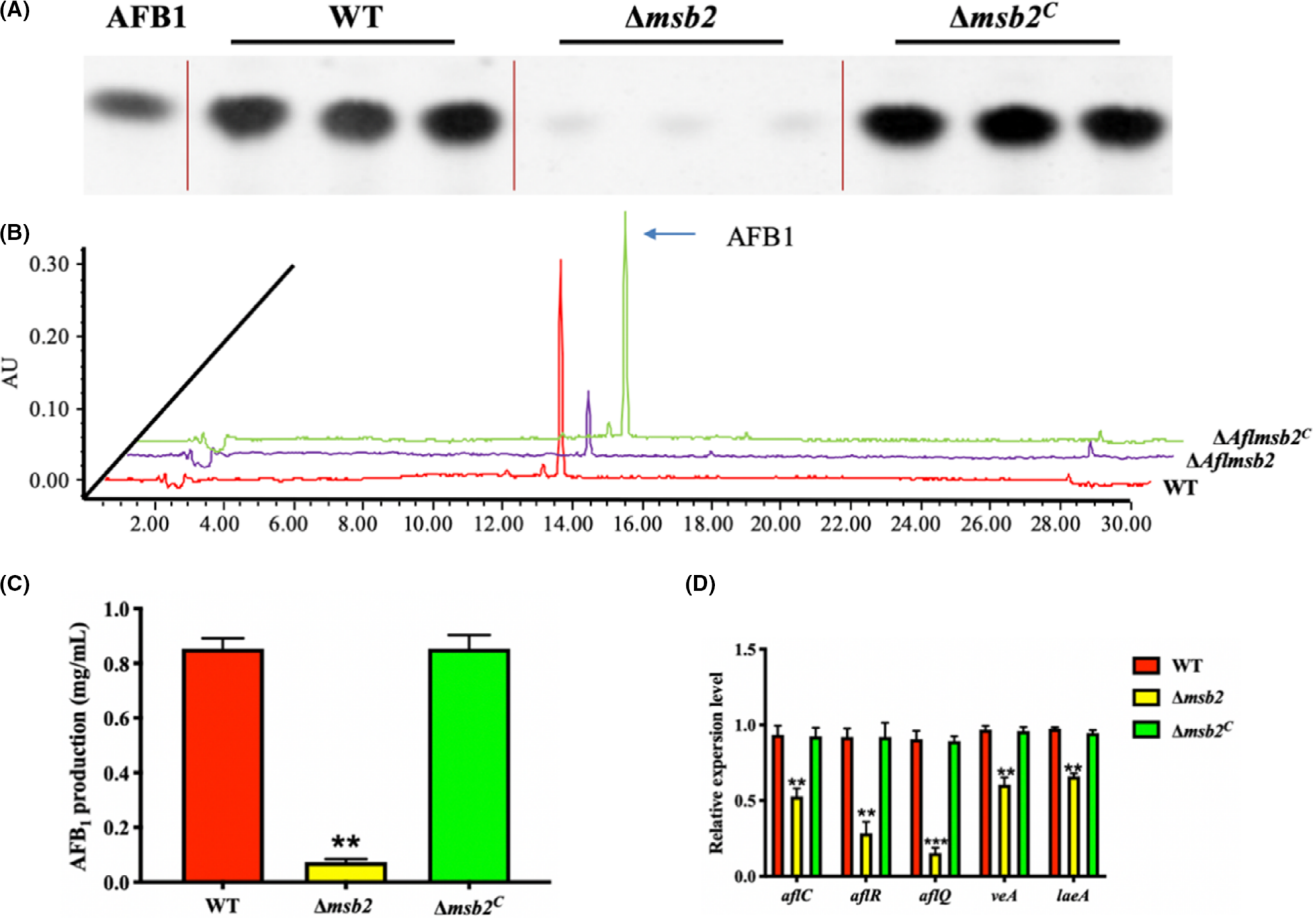
Analysis of aflatoxin production. A. Aflatoxin was detected by TLC after 7 days incubation on YES liquid medium at 29°C in dark. B. Analysis of aflatoxin production in WT, Δ*msb2* and Δ*msb2^C^* strains by HPLC. C. Quantification of AFB_1_ production as in (A). D. Transcript levels of *aflC*, *aflR*, *aflQ*, *veA* and *laeA* genes from WT, Δ*msb2* and Δ*msb2^C^* strains. ** and *** denotes *P* < 0.01 and *P* < 0.001, respectively.

*A. flavus* is notorious for its ability to produce aflatoxins (AFs), which are one of the most toxic and carcinogenic natural contaminants (Amare and Keller, 2014). In order to determine the specific function of Msb2 in AFs biosynthesis, WT, Δ*msb2* and Δ*msb2^C^* strains were cultured in liquid YES medium for 7 days, and AF production was tested by TLC and HPLC, respectively. TLC results indicated that AFB_1_ production in Δ*msb2* mutant strains was significantly decreased when compared to that of the WT and Δ*msb2^C^* mutants (Fig. 5A and C). Quantitative analysis by HPLC further confirmed that AFB_1_ production was down‐regulated in Δ*msb2* mutants (Fig. 5B). Additionally, to explore the possible reasons for the resultant decrease in AF production in Δ*msb2* mutant strain, we detected transcript levels of aflatoxin biosynthesis related genes (*aflC*, *aflR* and *aflQ*) and secondary metabolism regulator genes (*veA* and *laeA*) (Cary *et al*., 2015). The results revealed that the expression levels of *aflC*, *aflR*, *aflQ*, *veA* and *laeA* were all decreased significantly in Δ*msb2* in comparison to Δ*msb2^C^* and WT strains (Fig. 5D). In overall, these data demonstrated that Msb2 was involved in the regulation of aflatoxin biosynthesis in *A. flavus* through dominating the transcription of global regulators and aflatoxin‐producing related genes.

### Msb2 contributes to the pathogenicity

**Fig. 6:**
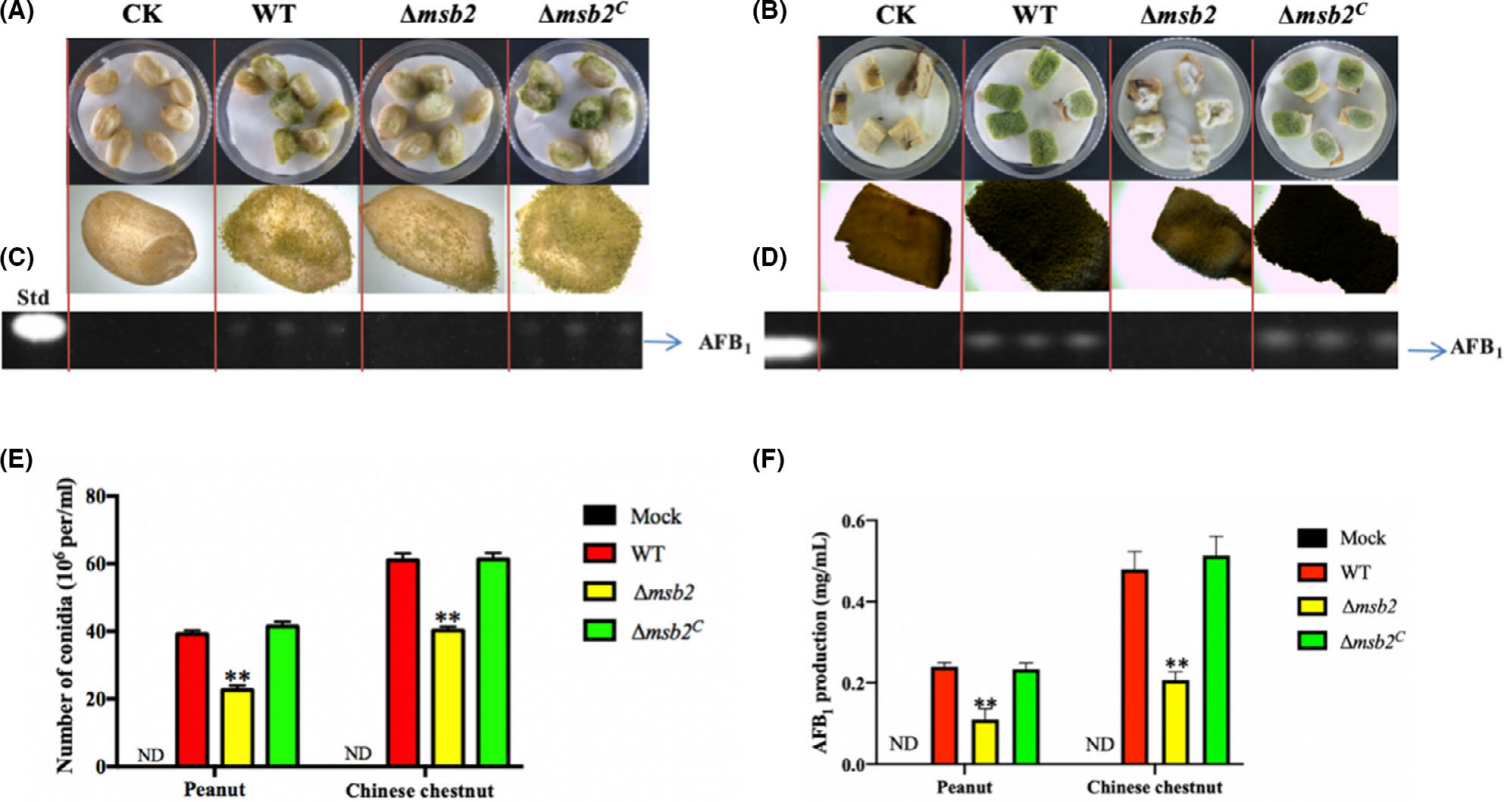
Pathogenicity of the WT, Δ*msb2* and Δ*msb2^C^* strains on plant seeds. A. Morphology of all strains grown on peanuts seeds at 29°C for 6 days. B. Morphology of all strains grown on Chinese chestnut at 29°C for 6 days. C. TLC measurements of AFB_1_ extracted from peanuts seeds. D. TLC measurements of AFB_1_ extracted from Chinese chestnut. E. Conidia production on peanuts and Chinese chestnut shown in A and B. F. Quantification analysis of AFB_1_ shown in C and D. **: *P* < 0.01. Std: standard AFB_1_.

Since *msb2* gene exhibited a variety of functions in growth, asexual conidia production, sclerotia formation and AFB_1_ biosynthesis, we anticipated that *msb2* might influence strain colonization on plant seeds. So peanut seeds and Chinese chestnut were inoculated with the WT, Δ*msb2* and Δ*msb2^C^* mutant strains. Visually, the WT and Δ*msb2^C^* strains showed stronger ability to infect and sporulate on peanut and Chinese chestnut surface in comparison to Δ*msb2* mutant (Fig. 6A and B). Then, the amount of conidia in the infected seeds was measured, and we found that conidia number was significantly decreased in Δ*msb2* mutant compared to Δ*msb2^C^* and WT (Fig. 6E). Further determination of AFB_1_ production from the infected seeds by TLC indicated that the AFB_1_ production in Δ*msb2* mutant was also significant reduced as compared with the WT and Δ*msb2^C^* mutant (Fig. 6C, D and F). These data confirmed that *msb2* is crucial for *A. flavus* maintaining full pathogenicity to plant seeds.

### Msb2 positive regulates phosphorylation of Hog1 in response to osmostress

**Fig. 7:**
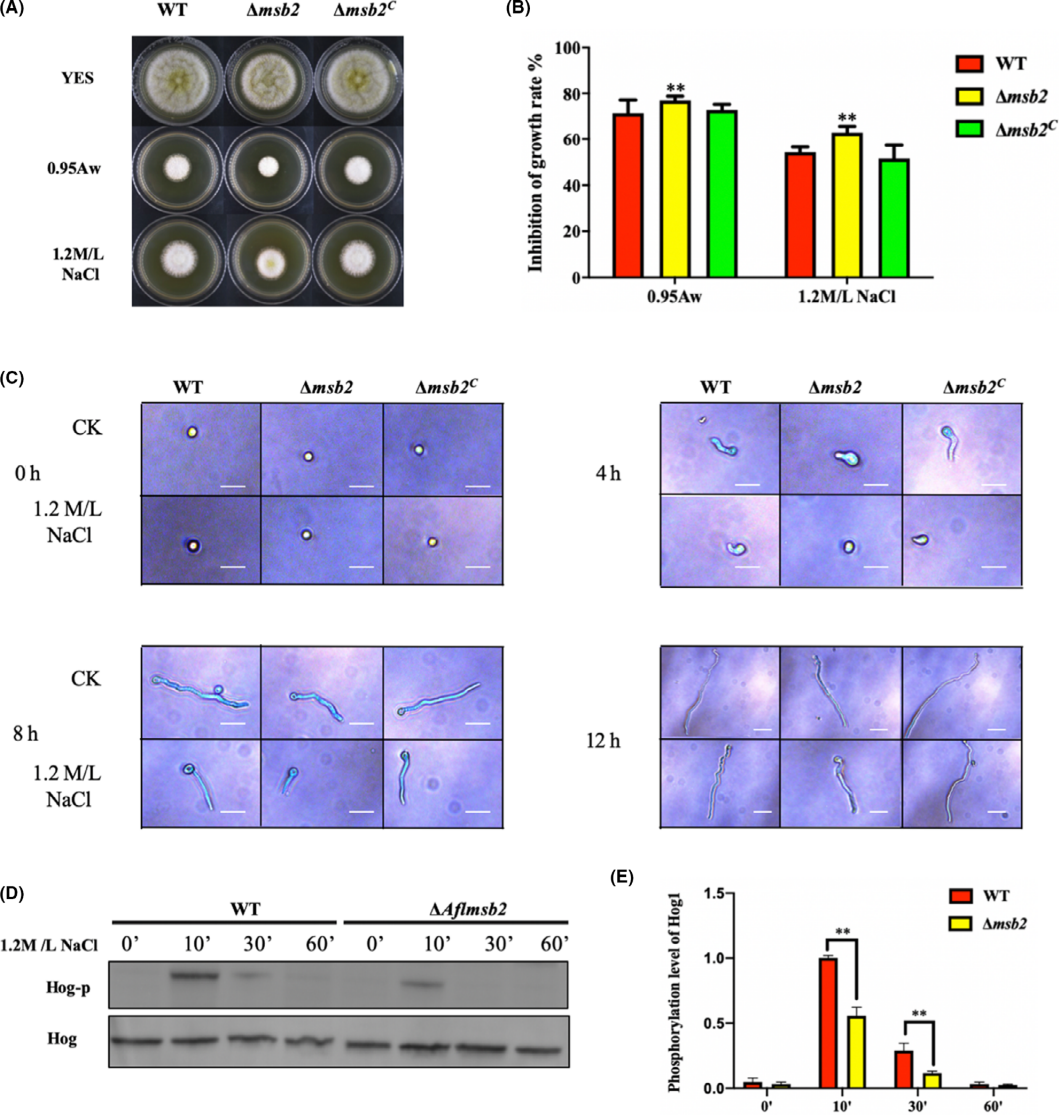
Deletion of *msb2* affects osmotic stress response in *A. flavus*. A. Colony morphology of the WT, Δ*msb2* and Δ*msb2^C^* strains cultured on YES medium under osmotic stress (0.95Aw and 1.2 M l^−1^ NaCl) at 37°C for 4 days. B. Growth inhibition rate of WT, Δ*msb2* and Δ*msb2^C^* strains was calculated based on A. C. Microscopic observation of WT, Δ*msb2* and Δ*msb2^C^* strains on YES liquid medium with osmotic stress (1.2 M l^−1^ NaCl) (bars = 50µm) . D. Phosphorylation levels of Hog1 in strains were measured by phosphor‐p38 MAPK antibody under NaCl stress, while Hog protein was used as loading control. E. Optical density semi‐quantitative analysis of the phosphorylation levels of Hog1 in the WT and Δ*msb2* strains. **, denotes *P* < 0.01.

Previous studies have reported Msb2 protein as a part of HOG‐MAPK pathway, played roles in various stress responses in fungi (Puri *et al*., 2012; Gurgel *et al*., 2019). Due to the osmosensor role of Msb2 in signal transduction in *S*. *cerevisiae* (Saito and Posas, 2012), we are especially interested in the role of AflMsb2 when facing hyperosmotic pressure. Then, osmotic stress (water activity, 0.95Aw and NaCl, 1.2M/L) were used in the YES solid medium, and the results showed that Δ*msb2* mutant was more sensitive to the osmotic stress when compared to Δ*msb2^C^* mutant and WT (Fig. 7A and B). Furthermore, cell growth upon high osmotic stress was also significantly inhibited in Δ*msb2* strain (Fig. 7C). After 4 h culture, defect in conidia germination was found in Δ*msb2*, and more severe situation appeared in the group which treated with NaCl (400× magnification). At 8 and 12 h, growth of Δ*msb2* was also obviously inhibited upon high osmotic stress (Fig. 7C). To confirm the relationship between *Aflmsb2* and HOG‐MAPK pathway, we detected the phosphorylation levels of Hog1 kinase in the WT and Δ*msb2* mutant under osmotic stress (1.2 M l^−1^ NaCl) at different times (0, 10, 30, 60 min). The WT and Δ*msb2* strains showed increased Hog1 phosphorylation after exposed to 1.2 M l^−1^ NaCl for 10 min. However, the phosphorylation level of Hog1 in Δ*msb2* mutant was lower than that in the WT. At 30 min, phosphorylation levels of Hog1 were both decreased in the WT and Δ*msb2* mutant as compared to 10 min, but the Hog1 phosphorylation in WT was still higher than that in the Δ*msb2* mutant (Fig. 7D). The protein expression levels of Hog1 were determined in the WT and Δ*msb2* mutant as well, and the result showed that Hog1 expressed no difference among these indicated strains under different conditions (Fig. 7D and E). In overall, these results suggested that AflMsb2 plays an important role in osmotic stress and involves in the phosphorylation of Hog1 in response to osmotic stress.

### Msb2 involves in CWI pathway

**Fig. 8:**
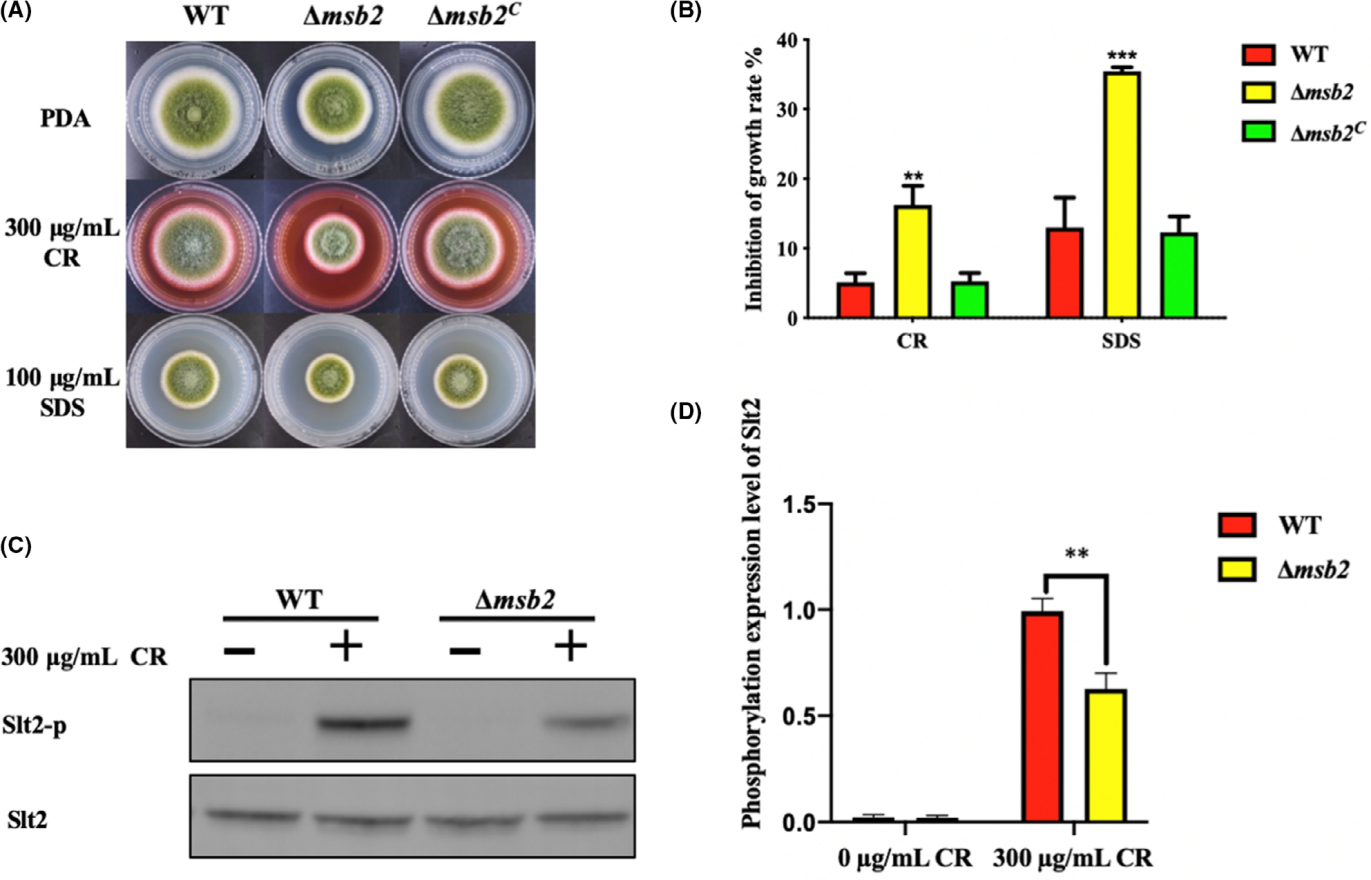
Deletion of *msb2* affects cell wall stress response in *A. flavus*. A. Colony morphology of WT, Δ*msb2* and Δ*msb2^C^* strains cultured on PDA medium with stress agents (300 μg ml^−1^ CR and 100 μg ml^−1^ SDS) at 37°C for 4 days. B. Inhibition rate of growth was calculated based on A. C. Phosphorylation levels of Slt2 were measured under stress (300 μg ml^−1^ CR), while Slt2 protein was used as loading control. D. Optical density semi‐quantitative analysis of the phosphorylation level of Slt2 in the WT and Δ*msb2* strains. ** and *** denotes *P* < 0.01 and *P* < 0.001, respectively.

CWI‐MAPK pathway was first discovered and identified in yeast, for it responses to cell wall stress maintaining normal life activities (Sanz *et al*., 2017). Slt2, MAP kinase, is the core component of CWI pathway which transmits a signal to the nucleus when cell surface sensors perceive the cell wall stress (Sanz *et al*., 2018). To explore the function of Msb2 in CWI, we cultured all strains on PDA media supplemented with cell wall‐damaging agent Congo red (CR) and cell membrane inhibitor Sodium Dodecyl Sulfate (SDS). The results showed that the growth inhibition rate of Δ*msb2* mutant in different additive media was significantly increased as compared to that of the WT and Δ*msb2^C^* mutant (Fig. 8A and B). To test the phosphorylation level of Slt2, WT and Δ*msb2* mutant were cultured in YES liquid media and treated with CR for 15 min. Western blotting results showed that the levels of phosphorylated Slt2 in indicated strains were significantly increased when induced by CR. However, there is a lower Slt2 phosphorylation level in Δ*msb2* strain than that in the WT under the stress (Fig. 8C and D), suggesting that Msb2 is able to impact the phosphorylation of Slt2 and involves in CWI pathway.

### Msb2 mutant has cell wall defects

**Fig. 9:**
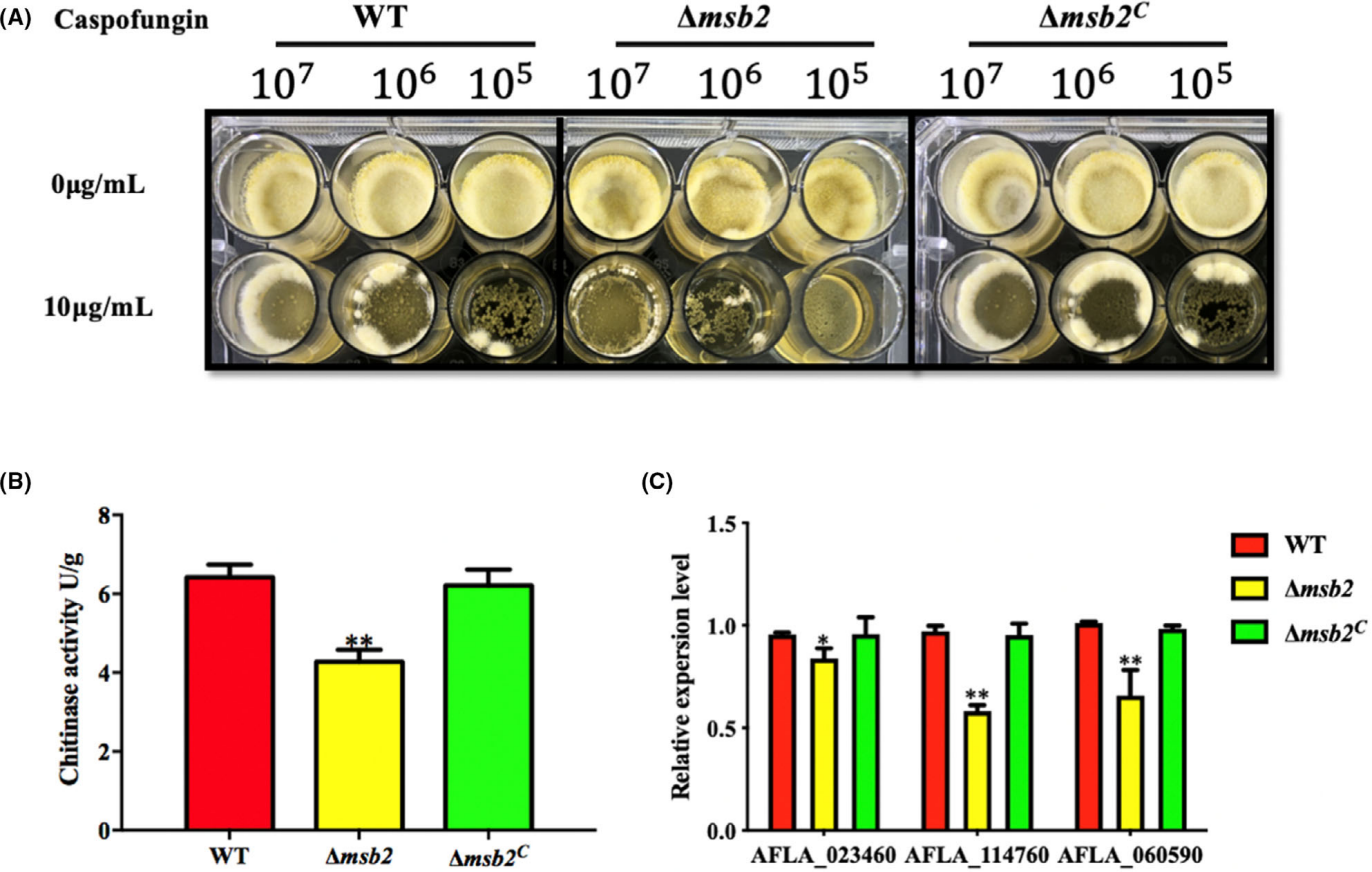
Deletion of *msb2* defects the cell wall integrity in *A. flavus*. A. WT, Δ*msb2* and Δ*msb2^C^* strains were grown on YES liquid media with caspofungin (10 μg ml^−1^) and cultured at 37°C for 72 h. B. Chitinase activity was measured in WT, Δ*msb2* and Δ*msb2^C^* strains of *A. flavus*. C. qRT‐PCR analysis of the expression levels of *β*‐1,3‐glucan and chitin synthase related genes (AFLA_023460 = *Aflags1*, AFLA_114760 = *AflchsB*, AFLA_060590 = *AflchsG*). * and ** denotes *P* < 0.05 and *P* < 0.01, respectively.

The cell wall components mainly contain *β*‐glucan and chitin in fungi (Gow *et al*., 2017). Δ*msb2* mutant from *A. flavus* exhibits hypersensitivity to cell wall stress, so we suspected that Msb2 may have some effects on glucan synthase and chitinase activity. To test these hypotheses, all strains were cultured on YES liquid media supplemented with caspofungin which is an inhibitor of *β*‐1,3‐glucan synthase. As shown in Figure 9A, the Δ*msb2* mutant was more sensitive to caspofungin when compare to the WT and Δ*msb2^C^*. Interestingly, the chitinase activity of Δ*msb2* mutant was also significantly decreased (Fig. 9B). Then, the expression levels of two chitin synthase genes (AFLA_114760 and AFLA_060590) and one *β*‐glucan synthase gene (AFLA_023460) were quantified. Under CR stress, the expression levels of three relative genes in Δ*msb2* mutant were markedly reduced in comparison with that in the WT and Δ*msb2^C^* (Fig. 9C). The aforementioned results suggested that Msb2 plays a crucial role in maintaining cell wall integrity.

## Discussion

MAPK cascades are essential for signal transduction in diverse eukaryotes including yeast and other fungi (Martínez‐Soto and Ruiz‐Herrera, 2017). HOG is one of the best understood MAPK pathways in yeast which play important roles in response to the stimuli from outside environments (Saito and Posas, 2012). The mucin‐like protein Msb2 is located on the cell membrane, acting as an osmosensor upstream the HOG pathway in *S. cerevisiae* (Tatebayashi *et al*., 2007). In the plant and animal pathogenic fungus *A. flavus*, our previous works suggested that three kinases of the HOG pathway play crucial roles in plant infection, aflatoxin biosynthesis and morphogenesis (Yuan *et al*., 2018; Tumukunde *et al*., 2019). But the role of AflMsb2 which has a typical transmembrane domain was yet to be identified (Fig. 1). In this study, we committed to investigate the biological function of Msb2 in *A. flavus*.

Msb2 is important for vegetative growth in many filamentous fungi. In *A. fumigatus*, MsbA is required for the conidiation and hyphal growth (Gurgel *et al*., 2019), and the homologous protein MsbA in *A. nidulans* regulates not only conidiation process but also vegetative growth (Brow *et al*., 2014). In *Cryptococcus neoformans* and *C. albicans*, Msb2 plays essential roles during vegetative growth (Román *et al*., 2009; So *et al*., 2018). The current study demonstrated retarded growth, and a substantial reduction in conidial production in Δ*msb2* mutant in comparison to the WT. Following the similar trend, the expression levels of key regulator genes *brlA* and *abaA*, which control asexual development, were also markedly reduced. In addition to conidiation, Msb2 also engages in sclerotia formation that helps the fungus survival in adverse environments. The sharp decrease of sclerotia formation in Δ*msb2* mutant was also verified by the down‐regulation of *sclR* and *nsdC* (Fig. 4). Interestingly, qRT‐PCR results also showed the decreased expression level of *veA* in ∆*Aflmsb2* in different culture conditions. In filamentous fungus *A. niger*, *veA* regulated conidia production through the development gene *brlA* which mediates the budding growth of conidiophores (Zhang *et al*., 2018a, 2018b,2018a, 2018b). As a global regulator, *veA* is necessary for sclerotia formation in *A. flavus* (Cary *et al*., 2007; Duran *et al*., 2007). Therefore, it could be speculated that Msb2 regulates conidial and sclerotia formation related genes, further effluences the conidia and sclerotia production in *A. flavus*.

Secondary metabolites synthesis in filamentous fungi is complicated (Yu *et al*., 2004a, 2004b,2004a, 2004b). In *S. cerevisiae*, Msb2 regulated the activity of p21‐activated protein kinases (PAKs) Ste20 and Cla4 (Tanaka *et al*., 2014). Similarly, the report from previous study further verified that the loss of PAKs *Aflste20* would lead to the impaired colonization and reduced AFB_1_ production (Li *et al*., 2019). It hints that genes in HOG pathway are important for AFB_1_ production in *A. flavus*. In this study, the AFB_1_ production was significantly decreased in Δ*Aflmsb2* mutant. To further study the mechanism of *Aflmsb2* in AFB_1_ synthesis, we also detected the expression levels of structural genes (*aflC* and *aflQ*) and regulatory gene (*aflR*) in aflatoxin biosynthesis gene cluster, and we found that they were all down‐regulated in Δ*Aflmsb2* mutant (Fig. 5). Based on the functional genomics and transcriptome analysis of *A. flavus*, the results reveal that *veA* regulated secondary metabolite gene clusters (Cary *et al*., 2015, 2007). Especially, *veA* controls aflatoxin synthesis cluster and directly impact the *aflR*, *aflD*, *aflM* and *aflP* transcripts (Duran *et al*., 2007). Previous study also showed that both *laeA* and *veA* genes are required for AFB_1_ production in *A. flavus* (Kale *et al*., 2008). In this study, expression levels of *laeA* and *veA* in ∆*Aflmsb2* were both significantly lower than that in WT. Therefore, we conclude that *msb2* is involved in AFB_1_ production and regulation of aflatoxin synthesis gene expression in *A. flavus,* most likely through *veA* and *laeA*.

Plant infection is a complex process and changes in the composition of cell wall of pathogenic fungus can reduce its ability to penetrate plant tissues (Geoghegan *et al*., 2017). In *A. flavus*, pathogenicity defect of *msb2* mutant occurred in seeds infection experiments, and the growth retardation and decreased conidiation in Δ*Aflmsb2* mutant could explain the defect of pathogenicity. On the other hand, the defective in cell wall integrity may strongly affect the infection ability of Δ*Aflmsb2*. In *A. fumigatus* and *A. nidulans*, it also showed the greatest reduction in mortality rate of mice for *msb2* deletion mutant compared to that for WT (Brow *et al*., 2014; Gurgel *et al*., 2019). *msb2* deletion mutant of *U. maydis* showed reduced colonization and appressoria in the plant surface (Lanver *et al*., 2010). It also reported that the mucin protein Msb2 is a broad range protectant against antimicrobial peptides in *C. albicans* (Swidergall *et al*., 2013). All these results indicated the important roles of *msb2* in self‐protection and pathogenicity in fungi.

In cells, genes change expression models to adapt the rapidly varying in environmental conditions (Karpinets and Foy, 2005). MAPK cascades regulate many biological processes and the most striking aspect is the role of MAP kinases (Lee *et al*., 2016). Msb2, as an osmosensor, was already identified in *S. cerevisiae* (Tanaka *et al*., 2014). In *C. albicans*, Msb2 can regulate adaptation to thermal stress, cell wall stress, oxidative stress and other stimuli (Román *et al*., 2009; Puri *et al*., 2012; Swidergall *et al*., 2013). Our results showed that under osmotic stress, Δ*msb2* mutant became more sensitive and had decreased phosphorylation level of Hog1 than WT (Fig. 7). The similar results were also occurred in *C. albican* and *F. oxysporum*, but needs hyperosmotic stress (minimum 1.5 M l^−1^ NaCl) (Pérez‐Nadales and Di Pietro, 2011; Puri *et al*., 2012). We also noticed that the phosphorylation of Hog1 in *C. neoformans msb2* deletion mutant was not affected when responding to osmotic shock (1 M l^−1^ NaCl). This outcome could be as a result that *sho1* has an overlapping function with *msb2* in *C. neoformans* HOG pathway (So *et al*., 2018). Surprisingly, at least five osmosensors (Msb2, Sho1, Sln1, Opy2 and Hkr1) were found to adapt hyperosmolarity in *S. cerevisiae*. There results suggested that Msb2 is important for full activation of the HOG pathway and positively regulate the Hog1 phosphorylation in *A. flavus*. But the exact role of Msb2 in the HOG pathway signal transduction needs further investigation.

The cell wall of fungi has a crucial role in cell division and hyphal development (Geoghegan *et al*., 2017). It has been reported that Msb2 homologues in *A. nidulans*, *C. albican* and *F. oxysporum* regulated the Slt2 (Mkc1) phosphorylation which was important for the cell wall biogenesis (Román *et al*., 2009; Brow *et al*., 2014; Perez‐Nadales and Di Pietro, 2015). Therefore, we are not surprised that Δ*Aflmsb2* mutant exhibited increased sensitivity to CR, SDS and caspofungin (Figs 8 and 9). As expected, the expression levels of cell wall chitin synthase genes and *β*‐glucan synthase gene were significantly decreased in Δ*Aflmsb2* mutant. The CR‐induced Slt2 phosphorylation level was also reduced in the Δ*Aflmsb2* mutant, indicating that AflMsb2 positively regulates the phosphorylation level of Slt2 in *A. flavus*. At the same time, our results also showed the reduced chitinase activity in Δ*Aflmsb2* mutant of *A. flavus*. Similarly, in *A. fumigatus*, MsbA positively modulates expression level of the chitin synthases (*chsA*, *chsB*), *β*‐1,3‐glucan synthase (*fksA*) and other related genes in the CWI pathway (Gurgel *et al*., 2019). MsbA interferences the chitin production in the cell wall and biofilm formation in *A. nidulans* (Brow *et al*., 2014). Interestingly, not only Δ*msb2* mutant but also Δ*sho1* mutant has cell wall defects in *C. albican*. Collectively, AflMsb2 plays a positive role in the activation of the phosphorylation of Slt2 and is important for cell wall biogenesis.

Overall, these results lead us to understand the role of *msb2* in reproduction, aflatoxins biosynthesis and pathogenicity in *A. flavus*. Our findings also provide a novel insight into the controlling of AF production and *A. flavus* in oil crops and food during storage.

## Experimental procedures

### Strains and media

*Aspergillus flavus* CA14 PTS (Δ*ku70*Δ*pyrG,* uracil auxotrophic) (Chang *et al*., 2010) was used as the parental strain for transformation. For *pyrG* auxotroph, the solid medium was supplied with uracil and uridine (1 g l^−1^ for each). The strains in this study were cultured on yeast extract‐sucrose (YES), minimal medium (MM), potato dextrose agar (PDA), glucose minimal medium (GMM) and complete medium (CM) for conidiation and mycelia growth assays, respectively (Ren *et al*., 2019). The sclerotia‐inducing Wickerham (WKM) medium was used for sclerotia assays. YES liquid medium was used for AFB_1_ assays. Each experiment was repeated at least 3 times.

### Domain and phylogenetic tree analysis

BLAST was carried out with the *S. cerevisiae* Msb2 protein sequence (NP_011528.3) to obtain *A. flavus* Msb2 (XP_002385498.1) and other *Aspergill* sequence. mega 7.0 software was used to create the phylogenetic tree. The protein domains were predicted by smart (http://smart.embl‐heidelberg.de/) and drawn by ibs 2.0 software (http://dog.biocuckoo.org/).

### Construction of mutant strains

The method of homologous recombination was used to construct *msb2* knockout mutant (Δ*Aflmsb2*). The primers used in this study were listed in Table 1. Double‐joint polymerase chain reaction (PCR) method (Yu *et al*., 2004a, 2004b,2004a, 2004b) was used to generate gene‐deletion cassettes which amplified by specialized primers. Then, overlap PCR products were transformed into the *A. flavus* CA14 PTS protoplasts. Positive transformants were screened by diagnostic PCR and Southern blot (Thermo Fisher Scientific, Waltham, MA, USA). To construct the Δ*Aflmsb2* complement strain (Δ*msb2^C^*), similar strategy was used and the *pyrG* marker was replaced by pyrithiamine resistance (*prtA*) marker. Then the gene complement cassettes were re‐introduced into Δ*Aflmsb2* protoplasts. The positive transformants were screened by PCR.

**Table 1:** PCR primer sets used in this study.

Primer	Sequence (5′–3′)	Characteristics
*msb2*‐P1	TCCTCCAGGCACGCAACA	For amplifying 5′UTR of *msb2* in Δ*msb2*
*msb2*‐P3	GGGTGAAGAGCATTGTTTGAGGC GACTCGGGGCGAAAGAGC	
*pyrG*‐F	GCCTCAAACAATGCTCTTCACCC	For amplifying *A. fumigatus pyrG*
*pyrG*‐R	GTCTGAGAGGAGGCACTGATGC	
*msb2*‐P6	GCATCAGTGCCTCCTCTCAGAC CTCTTTGGGACGAGGGTC	For amplifying 3′UTR of *msb2* in Δ*msb2*
*msb2*‐P8	CTCGATGTGATCCACCTAC	
*msb2‐*P2	GGAGATTGAAGCGGTGATA	For fusion PCR of Δ*msb2*
*msb2*‐P7	CATGGTGAAATACTCGGGAC	
*msb2*‐P9	GACTATCTCCATCAGCATCC	For ORF verification
*msb2*‐P10	GCGACGGTAGCGATATAG	
*msb2*‐qRT‐P9	CTCTGTTGTCCTCTATTCTGT	
*msb2*‐qRT‐P10	GGATGCTGATGGAGATAGTC	
*pyrG*‐907‐F	ATGACGGCGATGTAGGGA	For Δ*msb2* mutant screening
*pyrG*‐919‐R	CGACATCCTCACCGATTTCA	
*msb2*‐*C*‐P1	TGACGCCAGCGGTATTT	For amplifying 5′UTR of Δ*msb2^C^*
*msb2*‐*C*‐P3	CGAGGTGCCGTAAAGCACTAA TTAGTTCCATCCGAGGGAGTTTT	
*Ptr*‐F	TTAGTGCTTTACGGCACCTCG	For amplifying *PtrA*
*Ptr*‐R	ACTTTATCCGCCTCCATCCAG	
*msb2‐C‐*P6	CTGGATGGAGGCGGATAAAGT GCCTCAAACAATGCTCTTCACCC	For amplifying 3′UTR of *msb2* in Δ*msb2^C^*
*msb2‐C‐*P8	GTCTGAGAGGAGGCACTGATGC	
*msb2‐C‐*P2	TCGTTGCTCACTCCCTCA	For fusion PCR of*Δmsb2* ^*C*^
*msb2‐C‐*P7	ACTTGCCGCATACTCTGG	
*msb2‐C‐*AP‐F	CAGTGGTGTCGTTCCTTC	For *Δmsb2* *^C^*mutant screening
*msb2‐C‐*AP‐R	TGGTTCTCAGTGGTGTCA	
*msb2‐C‐*BP‐F	GTCGCCGCATACACTATT	
*msb2‐C‐*BP‐R	TCGTCACATCAGCAGAGA	

### Mycelial growth, conidiation and sclerotia analysis

The phenotypes of the WT, Δ*msb2* and Δ*msb2^C^* strains were observed using different medium. Assays for mycelial growth, fungal conidia and sclerotia formation were carried out according to our previously described methods (Yuan *et al*., 2018). Each experiment was repeated at least 3 times.

### Stress assay

For the stress assays, 1 μl of conidial suspension (10^7^ conidial ml^−1^) were inoculated at the centre of the YES and PDA medium plates supplemented with the following agents: 1.2 M l^−1^ NaCl, 0.95% water activity (95Aw: glycerol was used as an osmostressor to adjust the water availability), 300 μg ml^−1^ CR, 10 μg ml^−1^ caspofungin and 100 μg ml^−1^ SDS, respectively. All the plates were incubated at 37°C for 4 days under dark condition, and the relative inhibition rates were calculated as thus: [(diameter of control colony – diameter of treatment colony)/diameter of control colony] × 100%. Each experiment was repeated at least 3 times. For detecting the inhibition of cell growth upon high osmotic stress, liquid YES medium and liquid YES medium supplement with 1.2 M l^−1^ NaCl were used to inoculate the same aliquots of conidial suspension. After 1–24 h inoculation at 37°C on shaker, Leica confocal SP8 microscope (Leica, Heidelberg, Germany) with a 20× objective was used to record the hyphae growth, and Laetophcnol cotton blue was used to make the hyphae morphology more clear. The experiments were repeated 3 times, using 3 independent batches of conidia.

### Aflatoxins analysis

For aflatoxins (AFs) production, 10 μl conidial suspension (10^7^ conidial ml^−1^) of WT, Δ*msb2* and Δ*msb2^C^* strains were inoculated into 7.5 ml YES liquid medium respectively and incubated in 29°C under dark condition for 7 days. AFs production was detected by thin‐layer chromatography (TLC) (Haiyang Chemical, Qingdao, China). For quantitative analysis of aflatoxins production, methanol was used to resuspend the aflatoxin extracts. After filtration (0.22 µm), the samples were analysed by high‐performance liquid chromatography (HPLC) (Waters, Milford, MA, U.S.A.) on a Mycotox™ column (Water, Milford, USA) at 42°C. After the column was equilibrated in running solvent (water:methanol:acetonitrile, 56:22:22), 10 µl samples were injected, and isocratic runs were conducted for 15 min in 100% running solvent at a flow rate of 1.0 ml min^−1^. Aflatoxins were checked using a fluorescent detector (Water, Milford, USA) with excitation and emission wave lengths of 365 and 455 nm, respectively. Each experiment was repeated 3 times.

### Seeds infections

The ability of WT, Δ*msb2* and Δ*msb2^C^* strains to infect plant seeds was assayed as described previously (Yang *et al*., 2019). Seeds were inoculated with conidial suspension and cultured at 29°C, then 700 μl sterile water was added to keep the filter paper almost moist. After 6 days, seeds were harvested in 50 ml centrifuge tubes (one plate corresponds to one centrifuge tube) which contained 15 ml sterile water and 7.5 μl Tween 20. In order to release conidia on seeds surface, tubes were vigorously mixed for 5 min. The number of conidia was counted and AF was extracted according to the method described above. Each experiment was repeated at least 3 times.

### Western blot analysis

The conidia (6 × 10^5^) of WT, Δ*msb2* and Δ*msb2^C^* strains were inoculated in YES liquid medium respectively and cultivated for 48 h. Whole‐cell extraction and Western blot were performed as described previously (Lan *et al*., 2019). Phospho‐p38 MAPK antibody (Cell Signaling Technology, Boston, MA, USA) and Anti‐phospho‐p44/42 MAPK (Erk 1/2) antibody (Cell signaling Technology, MA, USA) against target proteins were used to detect the specific phosphorylated proteins. Monoclonal antibody Hog1 (Santa Cruz Biotechnology, Dallas, Texas, USA) and anti‐AflSlt2 antibody (prepared by our laboratory) were used as loading controls respectively. Enhanced chemiluminescence (ECL) substrate was used for immunoblot analysis and chemiluminescence was measured by G‐BOX Chemi XT4 (Syngene, HK, China).

### Chitinase activity assay

The conidia (6 × 10^5^) of WT, Δ*msb2* and Δ*msb2^C^* strains were grown in YES liquid medium in a rotary shaker (180 r min^−1^) for 72 h. The mycelia were removed with glass filter, and ultrasonic crusher (Scientz, Zhejiang, China) was used for protein extraction. Chitinase activity was measured using a chitinase activity detection reagent (Solarbio Science, Beijing, China) as described by Yanai *et al*. (1992). Chitinase hydrolyses chitin to produce n‐acetylglucosamine, which is further combined with 3, 5‐dinitrosalicylic acid to produce brownish red compounds. There is a characteristic absorption peak at 540 nm detected by enzyme‐labelled instrument (Molecular Devices, San Jose, CA, USA), and the increase of absorption reflects the activity of chitinase. The experiment was repeated 3 times.

### Quantitative RT‐PCR analysis

For qRT‐PCR, the mycelia of WT, Δ*msb2* and Δ*msb2^C^* strains were harvested at WKM, PDA and YES medium at 37°C. The total RNA was got from 100 mg ground mycelium through the TRIzol reagent (Biomarker Technologies, Beijing, China), and the First‐strand cDNA was obtained by the cDNA Synthesis SuperMix (TransGen Biotech, Beijing, China). qRT‐PCR was performed on the Thermo Fisher Scientific Real‐time PCR System (Finland) using SYBR Green qPCR Mix (DongSheng Biotech, Guangzhou, China). The *actin* gene of *A. flavus* was utilized as the reference gene, and the relative expression of the target gene was calculated using the 2^−ΔΔ^ *^Ct^* method (Livak and Schmittgen, 2001). The qRT‐PCR primers were listed in Table 2. All assays of qRT‐PCR were conducted with technical triplicates for each sample, and each experiment was repeated at least 3 times.

**Table 2:** qRT‐PCR primer sets used in this study.

Primer	Sequence (5′–3′)	Characteristics
*abaA*‐ qRT‐F	TCTTCGGTTGATGGATGATTTC	For *abaA* qRT‐PCR
*abaA*‐ qRT‐R	CCGTTGGGAGGCTGGGT	
*brlA*‐ qRT‐F	GCCTCCAGCGTCAACCTTC	For *brlA* qRT‐PCR
*brlA*‐ qRT‐R	TCTCTTCAAATGCTCTTGCCTC	
*nsdC*‐ qRT‐F	GCCAGACTTGCCAATCAC	For *nsdC* qRT‐PCR
*nsdC*‐ qRT‐R	CATCCACCTTGCCCTTTA	
*sclR*‐ qRT‐F	CAATGAGCCTATGGGAGTGG	For *sclR* qRT‐PCR
*sclR*‐ qRT‐R	ATCTTCGCCCGAGTGGTT	
*aflC‐* qRT‐F	GTGGTGGTTGCCAATGCG	For *aflC* qRT‐PCR
*aflC‐* qRT‐R	CTGAAACAGTAGGACGGGAGC	
*aflR‐* qRT‐F	AAAGCACCCTGTCTTCCCTAAC	For *aflR* qRT‐PCR
*aflR‐* qRT‐R	GAAGAGGTGGGTCAGTGTTTGTAG	
*aflQ‐* qRT‐F	GTCGCATATGCCCCGGTCGG	For *aflQ* qRT‐PCR
*aflQ‐* qRT‐R	GGCAACCAGTCGGGTTCCGG	
*Ags1‐* qRT‐F	CTACGCCCGTTATCCCATCT	For *Ags1* qRT‐PCR
*Ags1‐* qRT‐R	TGACATCAAGACCAGCCCAT	
*ChsB‐* qRT‐F	CGCTAACTATCCGCAGAG	For *ChsB* qRT‐PCR
*ChsB‐* qRT‐R	CACCACGATGTTGATGAAG	
*ChsG‐* qRT‐F	AGGAGTTTACCCACATGCGA	For *ChsG* qRT‐PCR
*ChsG‐* qRT‐R	TTGAGGTTCACAATGTCGCG	
*actin*‐F	ACGGTGTCGTCACAAACTGG	For *actin* qRT‐PCR
*actin*‐R *veA‐*qPCR‐F *veA‐*qPCR‐R *laeA‐*qPCR‐F *laeA‐*qPCR‐R	GCGTATCGTCGTTACCTCATC TATCATTCCGTGGCTCAAT GAGAGGTACTGCTGGATG TTGTTGGGGTTGACCTTGCT GCCATCCCATCACACTTCCA	For *veA* qRT‐PCR For *laeA*‐qRT‐PCR

### Statistical analysis

graphpad prism 7 (https://www.graphpad.com) was used to analyse the statistics and significance. Student’s *t* test was performed for comparison of two different groups, while multiple groups comparisons were through One‐way analysis of variance (ANOVA) test.

## Conflict of interest

The authors declare that they have no known competing financial interests or personal relationships that could have appeared to influence the work reported in this paper.
